# Action of Monomeric/Gemini Surfactants on Free Cells and Biofilm of *Asaia lannensis*

**DOI:** 10.3390/molecules22112036

**Published:** 2017-11-22

**Authors:** Anna Koziróg, Dorota Kręgiel, Bogumił Brycki

**Affiliations:** 1 Institute of Fermentation Technology and Microbiology, Faculty of Biotechnology and Food Science, Lodz University of Technology, Wolczanska 171/173, 90-924 Lodz, Poland; dorota.kregiel@p.lodz.pl; 2 Laboratory of Microbiocides Chemistry, Faculty of Chemistry, Adam Mickiewicz University in Poznan, Umultowska 89b, 61-614 Poznań, Poland; brycki@amu.edu.pl

**Keywords:** gemini surfactants, antimicrobial activity, *Asaia*, antibiofilm agent, polypropylene

## Abstract

We investigated the biological activity of surfactants based on quaternary ammonium compounds: gemini surfactant hexamethylene-1,6-bis-(*N,N*-dimethyl-*N*-dodecylammonium bromide) (C6), synthesized by the reaction of *N,N*-dimethyl-*N*-dodecylamine with 1,6-dibromohexane, and its monomeric analogue dodecyltrimethylammonium bromide (DTAB). The experiments were performed with bacteria *Asaia lannensis*, a common spoilage in the beverage industry. The minimal inhibitory concentration (MIC) values were determined using the tube standard two-fold dilution method. The growth and adhesive properties of bacterial cells were studied in different culture media, and the cell viability was evaluated using plate count method. Both of the surfactants were effective against the bacterial strain, but the MIC of gemini compound was significantly lower. Both C6 and DTAB exhibited anti-adhesive abilities. Treatment with surfactants at or below MIC value decreased the number of bacterial cells that were able to form biofilm, however, the gemini surfactant was more effective. The used surfactants were also found to be able to eradicate mature biofilms. After 4 h of treatment with C6 surfactant at concentration 10 MIC, the number of bacterial cells was reduced by 91.8%. The results of this study suggest that the antibacterial activity of the gemini compound could make it an effective microbiocide against the spoilage bacteria *Asaia* sp. in both planktonic and biofilm stages.

## 1. Introduction

Acetic acid bacteria of the *Asaia* species are increasingly isolated from spoiled soft drinks in Europe [1,2,3]. Initially identified as an epiphytic bacteria that is associated with exotic plant species, including the orchid tree (*Bauhinia purpurea*) and plumbago (*Plumbago auriculate*) [2], *Asaia* spp. are able to grow well in nonalcoholic beverages, even in the presence of preservatives such as benzoate, sorbate, and dimethyl dicarbonate [1]. Due to their production of exopolysaccharides, these bacteria show exceptional ability to colonize the internal areas of production systems. Due to the hydrophilic nature, their cells are able to adhere to various types of surfaces that are used in the food industry and form biofilms [4,5]. In biofilm form, *Asaia* spp. cells are able to survive the conventional processes of washing and disinfection, making them difficult to eliminate in technological lines. A possible solution might be to find new antimicrobial agents that are more effective at eliminating the growth of these bacteria in production environments.

Quaternary ammonium compounds (QACs) are often used as cleaners and disinfectants in the food industry. They are typical surfactants, composed of a hydrophilic head and a hydrophobic tail. Quaternary ammonium compounds have a wide spectrum of ability to inhibit the growth of bacteria, yeasts, molds and viruses [6,7,8,9,10]. They are the most important group of microbiocides that are used in hygienization processes. However, numerous studies have shown increased microbial resistance to various QAC-based disinfectants [11,12,13,14,15]. In the present study, we therefore used gemini surfactants with superior surface-active properties than conventional monomeric surfactants.

Gemini surfactants (GS) are compounds that are composed of two hydrophilic head groups and two hydrophobic tails linked by a spacer at the head groups or closed to them.These compounds are characterized by very low critical micelle concentrations (CMC), surface tension (γ), and minimal inhibitory concentration (MIC) [16,17,18,19]. Due to their structure, dimeric surfactants lower surface tension more efficiently and have better foaming properties [16,20,21,22]. However, their effectiveness depends on their structure [23,24]. The most active GS against bacteria and microscopic fungi are those with 10–12 carbon atoms in the hydrophobic part. The influence of GS on microorganisms depends also on the spacer, their length, and chemical structure. Based on the results obtained by Zhang et al. [25] and Banno et al. [26] we can observe that a longer spacer in GS causes the better antimicrobial activity. The anion is also an important element of the molecule. Bromides are more effective than chlorides [27,28].

The purpose of this work was to determine the biological activity of gemini surfactant hexamethylene-1,6-bis-(*N,N*-dimethyl-*N*-dodecylammonium bromide (C6) and its monomeric analogue *n*-dodecyltrimethylammonium bromide (DTAB) against *Asaia lannensis* isolated from spoiled soft drinks.

## 2. Results

### 2.1. Minimal Inhibitory Concentration (MIC)

Minimal inhibitory concentration was used as a comparative measure of the effectiveness of hexamethylene-1,6-bis-(*N,N*-dimethyl-*N*-dodecylammonium bromide) (C6) and dodecyl(trimethyl) ammonium bromide (DTAB) against *A. lannensis* FMW 1. Table 1 presents the MICs for these two compounds in different culture media.

Gemini surfactant inhibited *A. lannensis* growth at 17–563 times lower concentrations in comparison to its monomeric analogue. Interestingly, the sensitivity of *A. lannensis* depended on the type of culture medium used. In general, a greater sensitivity was noted in minimal media. For DTAB, the highest MIC values were noted in rich culture medium TSB + 2% glucose and in minimal medium + 2% maltose (Table 1). In bacterial cultures in minimal media with gemini surfactant C6, the MIC values were the same, regardless of the type of carbon source. It is worth noting that the MIC of C6 in TSB + 2% glucose medium was half of that in minimal media. The MIC results for C6 are similar to those reported in our earlier studies (0.0036–0.0145 μM/mL) for *Pseudomonas aeruginosa*, *Staphylococcus aureus*, *S. epidermidis*, and *Brochothrix thermosphacta* [18,19]. Antimicrobial activity of GS of the 12-s-12 series on *Escherichia coli* and *Staphylococcus aureus* was previously examined by Zhang et al. [25]. The lowest MIC value was obtained for the compound containing 8 methylene group in the spacer. The compound of the 12-6-12 series inhibited the growth of *Escherichia coli* at a concentration of 86.8 µM, which is more than 24 times higher in comparison to the MIC for *Asaia lannensis*. The influence of cationic gemini surfactants on gram-negative bacteria was also reported by Paniak [23]. The MIC value for the compound of the 12-5-12 series equaled 1 µM (for *Escherichia coli*) and 2 µM (for *Pseudomonas aeruginosa*), while for 12-7-12, 2 and 8 µM, respectively. Those MIC values are similar as for acetic acid bacteria (MIC = 3.6 µM).

It is noteworthy that, when comparing the results that are presented by many authors [19,20,23,24,25,26], apart from the differences in chemical structure of surfactants, diverse species and even kinds of microorganisms should be considered. As stated by the author, the differences are related to a single species or strain [18]. Moreover, MIC value is affected by the environment. Our results confirm that the sensitivity of bacterial strains to surfactants depends on the environmental conditions. Choi et al. studied the effect of bacterial growth medium on MIC values measured for *E. coli* [29]. They suggest that certain components in the culture media may form various electrostatically-bound complexes with active antibacterial compounds. Culture media with highly complex components may therefore protect microorganisms from the presence of antimicrobial compounds and from conditions that might otherwise cause sudden, potentially damaging changes in bacterial cells.

### 2.2. Viability of A. lannensis Cells in the Presence of Microbiocides

The viability of the planktonic cells was analyzed in medium with the addition of surfactants.

**Figure 1:**
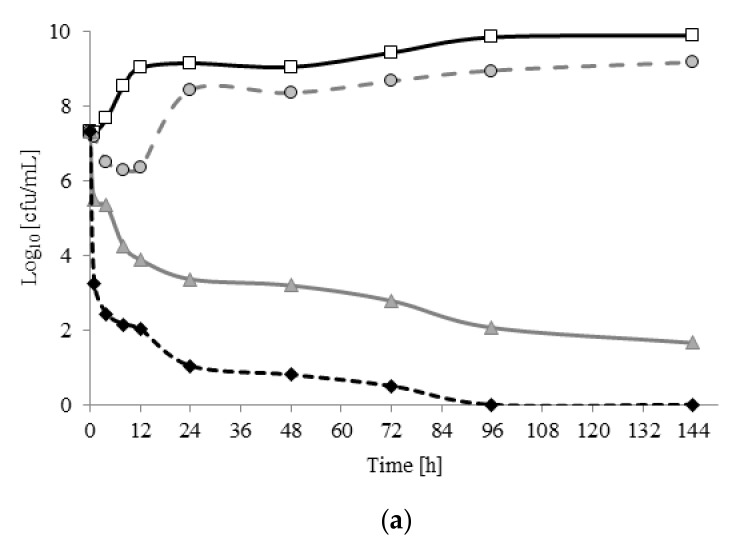

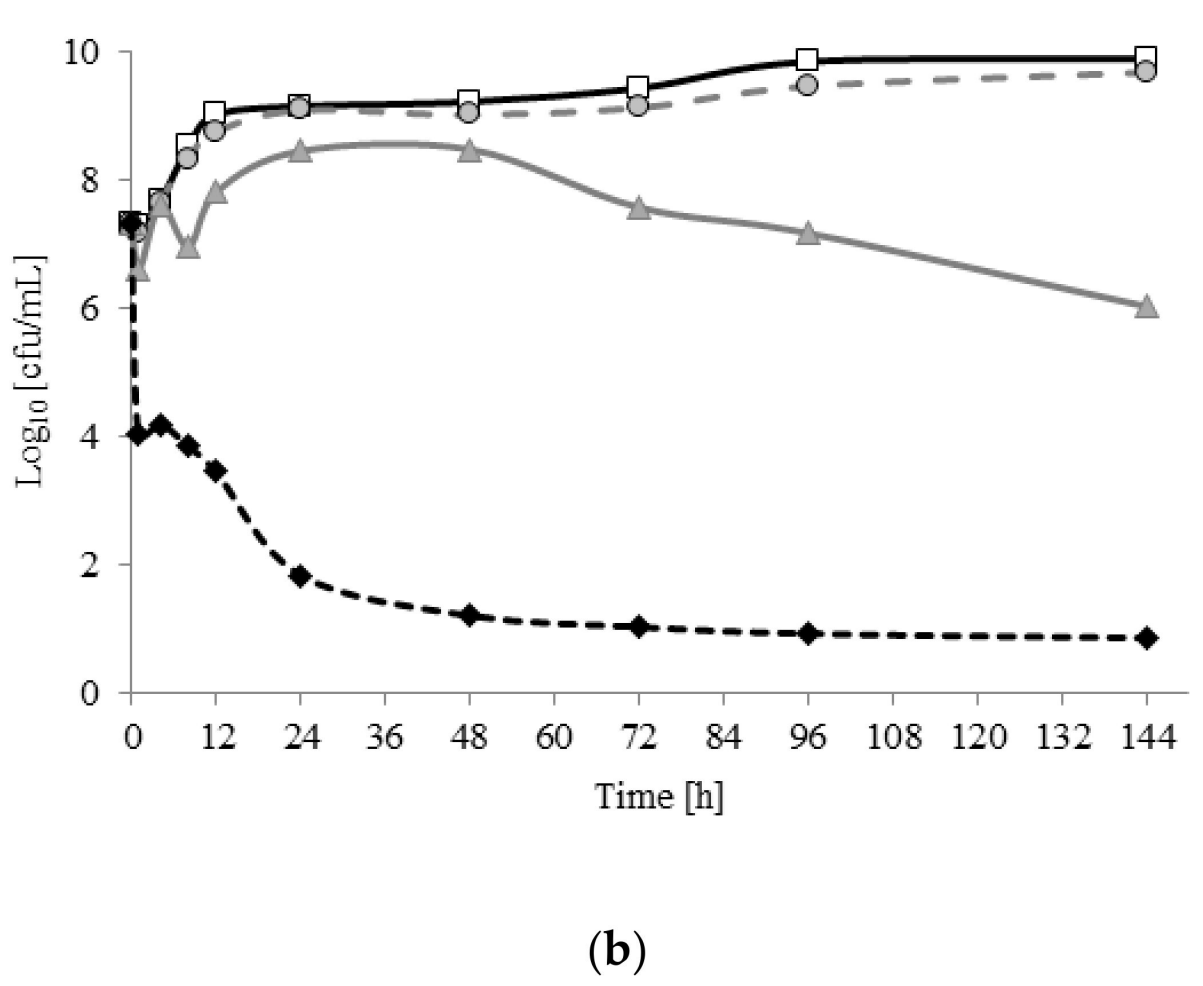
The growth of *A. lannensis* FMW1 in medium with microbiocides (**a**) hexamethylene- 1,6-bis-(*N,N*-dimethyl-*N*-dodecylammonium bromide) (C6) and (**b**) *N*-dodecyltrimethylammonium bromide (DTAB). The results are presented after cells treatment by surfactants in the concentration ¼ minimal inhibitory concentration (MIC) (dark grey circle marker), ½ MIC (clear grey triangle marker), MIC (black rhombus marker), and compared with the control sample -without the addition of surfactants (white square marker).

Figure 1 shows the number of viable bacterial cells (cfu/mL) after treatment with gemini and monomeric surfactant. Although it was 560 times less concentrated, gemini surfactant C6 caused a greater reduction in the number of bacterial cells than its monomeric analogues. After 24 h of incubation with gemini or monomeric surfactant at MIC, bacterial growth was inhibited, and the number of viable cells fell to ≤ 2 log. Similar results were obtained for *P. aeruginosa* and *S. aureus* in our earlier studies [18]. Houari et al. also noted sharp reductions in the number of viable *E. coli*, *K. pneumoniae*, *P. aeruginosa*, and *S. epidremidis* cells after 24 h of treatment with benzalkonium chloride (BAC) at MIC [30]. The MIC for BAC was higher (0.008–0.25 mg/mL) than that for gemini C6 (0.0024 mg/mL). Ortiz et al. report the MIC for BAC against the *Listeria monocytogenes* strain as 0.0025 mg/mL [31]. However, the number of viable cells after 24 h of treatment was > 4 log, falling to around 1.8 log at 2 MIC. After 24 h of incubaction, dimeric surfactant reduced the number of viable cells to 1.0 log and 3.4 log for MIC and ½ MIC, respectively. The action of DTAB only decreased the number of cells at MIC. At sub-MIC levels (½ and ¼ MIC for DTAB and ¼ MIC for C6) after 24 h of incubation, similar trends were observed in cultures without microbiocide (control) to those for *A. lannensis*.

Moreover, in the sample with DTAB at ½ MIC, increasing the period of incubation to 144 h caused a reduction in the numbr of viable bacterial cells to 6 log. No live bacterial cells were noted after 96 h of incubation with gemini surfactant C6 at MIC. In other samples, no notable changes were observed with an increased incubation time. This is interesting, especially as *Asaia* spp. bacteria have been reported as being as resistant to various conventional disinfectants and preservatives used in the food industry [1,32,33].

The concentration of antimicrobial agents and contact time are both very important parameters for the effective elimination of microbial cells in different environments. Applying bioactive compounds at insufficient concentrations (sub-MIC) may reduce the susceptibility of microorganisms, and over time lead to the formation of resistant forms [34]. Short contact times can reduce cell numbers, but the remaining cells may still be active.

### 2.3. Planktonic Growth and Biofilm Formation

**Figure 2:**
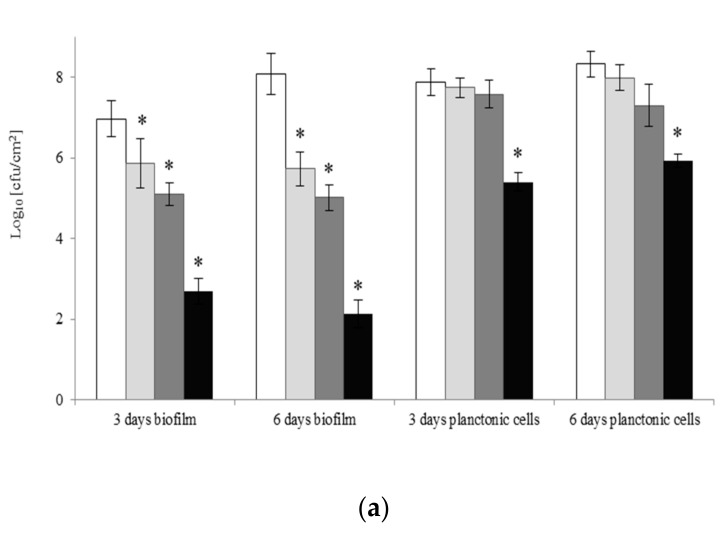

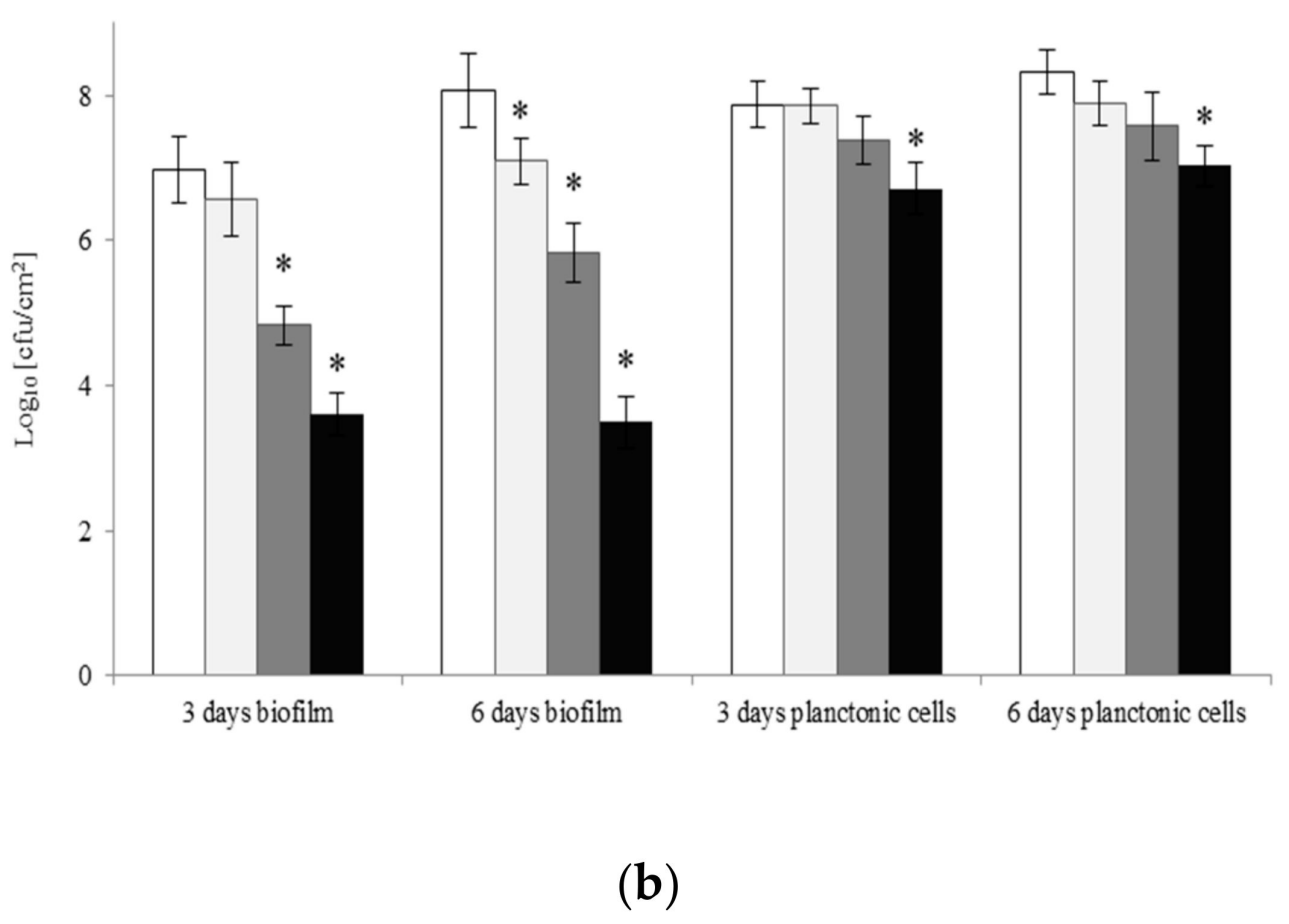
Effect of gemini C6 (**a**) and monomeric DTAB (**b**) surfactants on biofilm formation by *A. lannensis* FMW1. The results are presented after 1-h cells treatment by surfactants in the concentrations: ¼ MIC (clear grey bar), ½ MIC (dark grey bar), MIC (black bar), and compared to the control sample without surfactants (white bar). * Reduced value of log _10_ differed significantly from the control without surfactant.

Figure 2 shows planktonic growth and biofilm formation after treatment of *A. lannensis* with surfactants at different concentrations for 1 h. After brief treatment using monomeric or gemini surfactants at MIC, the number of viable planktonic cells decreased in comparison to the control samples (*p* < 0.05). However, by prolonging the duration of surfactant treatment, an increase in the number of viable bacterial cells was noted.

Changes in the planktonic cell counts in bacterial cultures with C6 or DTAB in concentrations of ¼ and ½ MIC were not statistically significant (*p* < 0.05), when compared to those for cultures without surfactants. Short-term exposure to the two surfactants resulted in a decrease in the number of cells capable of biofilm formation. In the culture medium with gemini C6 at MIC, the numbers of viable cells after three and six days of incubation were 2.7 logs and 2.1 logs, respectively. In samples with monomeric surfactant under the same conditions, the numbers of cells adhered to the polypropylene decreased to 3.6 logs and 3.5 logs. Both microbiocides also reduced biofilm formation at ½ and ¼ MIC (1–3 log). Only in the sample incubated for three days with DTAB at ¼ MIC was the difference between the test sample and control (without microbiocide) not statistically significant (0.2 log). Our results show that, despite the reductions in cell counts after 1 h with C6 (4 logs) and DTAB (3.3 logs) (Figure 1), survivors were able to grow and adhere to the polypropylene surface.

The ability of *Asaia* sp. bacteria to form biofilms on the propylene test surface was also investigated, in the presence of gemini C6 and DTAB. The bacterial cells were kept in contact with the biocidal compounds at concentrations of ¼ MIC, ½ MIC, and MIC for six days. These concentrations were used because they simulate conditions occurring inside the production lines, when the rinse step is not carried out after disinfection and the biocidal compound remains in the production environment. Sub-MIC concentrations of surfactant can allow live cells to survive on the surface, which may produce biofilm over time.

**Figure 3:**
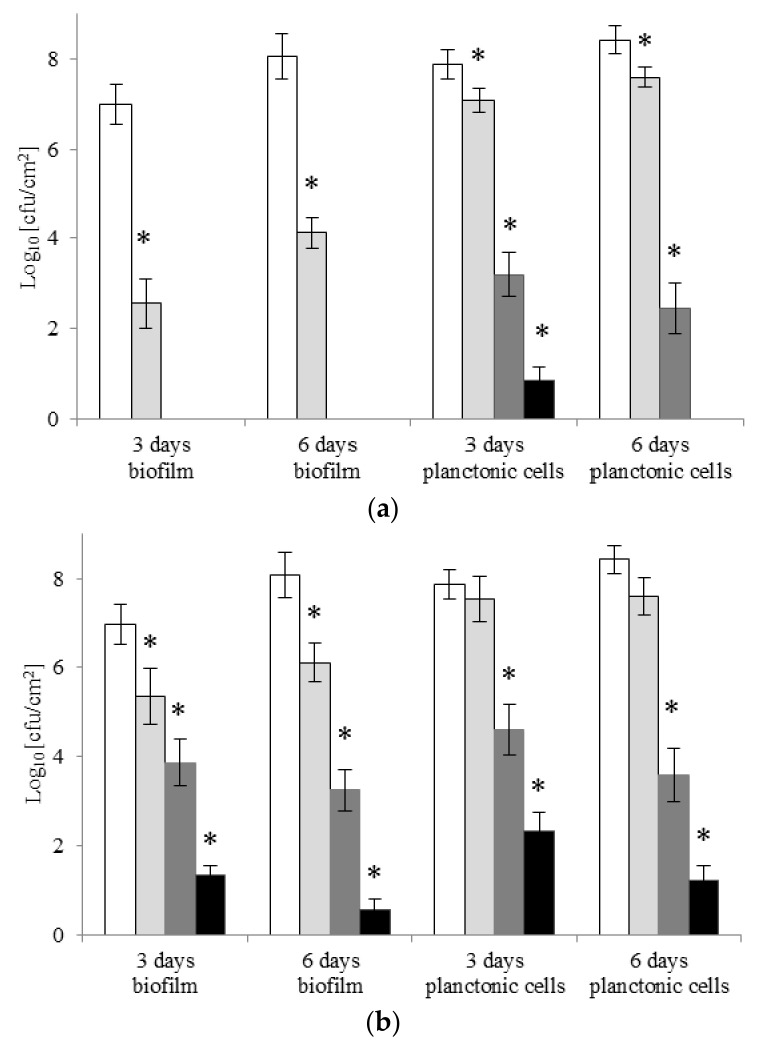
Effect of gemini C6 (**a**) and monomeric DTAB (**b**) surfactants on biofilm formation by *A. lannensis* FMW1. The results are presented for samples with surfactants in the concentration ¼ MIC (clear grey bar), ½ MIC (dark grey bar), MIC (black bar), and compared to the control sample without surfactants (white bar). * Reduced value of log _10_ differed significantly from the control without surfactant.

Figure 3 shows the effects of the two surfactants on biofilm formation by *A. lannensi*s. With C6 at MIC and ½ MIC concentrations, *A. lannensis* cells were unable to produce biofilm, although they survived in suspension as planktonic cells. After six days with C6 at MIC, the cells were also unable to survive in suspension. At ¼ MIC (0.0009 μM/mL), gemini surfactant also reduced the number of cells (both planktonic and adhered) in comparison to the control (*p* < 0.005). However, it was noted that after six days of incubation, the number of viable bacterial cells (both free and adhered) increased. Generally, the number of planktonic cells in samples with monomeric surfactant reduced in comparison to the control samples (*p* < 0.005). An exception was the sample after three days of incubation with DTAB at ¼ MIC. Longer treatment with DTAB did not completely inhibit cell adhesion on the tested polymer surface, but at MIC (2.026 μM/mL) the levels of inhibition were high (80.9 and 93.0%, after three and six days, respectively) (Table S1 in supplementary).

*Asaia* is a relatively newly discovered and unstudied genera of acetic acid bacteria [35]. There is no data in the literature on the effects of biocidal compounds on these spoiling microorganisms. Horsakova et al. tested *Asaia* spp. bacteria in the presence of various preservatives that are used in the beverage industry [1]. *A. lannensis* FMW1 has been shown to exhibit high adhesion abilities, particularly to polymer surfaces [5]. Fruit juices were found to lower the adhesive ability of *A. lannensis* FMW1, similarly to the surfactants that are presented in this work. Antolak et al. tested the antiadhesive properties of various high-polyphenolic fruit juices against *Asaia* spp. [32,33,36,37]. Ortiz et al. observed that BAC at MIC concentrations (0.01 mg/mL) and ½ MIC reduced the adhesion ability of *L. monocytogenes* S1 by 100% [31]. However, with the S11 strain, at an MIC of 0.0025 mg/mL, biofilm reduction was only 30%. The gemini surfactant (C6) that was used in the present study against the adhesion of *A. lannensis* was 100% effective at the very low concentration of ½ MIC, i.e., 0.0018 μM/mL (0.0012 mg/mL).

Essential oils are more and more commonly used as natural antibiofilm agents. One such oil is yarrow essential oil (YEO). Jadhav et al. investigated the ability of YEO at MIC = 3.13 (*v*/*v*) to inhibit the growth of *Listeria* spp. biofilm on polyethylene surfaces [38]. Growth inhibition of around 29% was reported after 24 h. Thus, at insufficient concentrations the application of either natural or synthetic microbiocides may contribute to biofilm formation, even during constant contact with cells. Nevertheless, our study shows that surfactants at MIC can exhibit very good antiadhesive properties against *A. lannensis* on polypropylene.

### 2.4. Eradication of Biofilm

In addition to preventing adhesion, biocidal compounds should be able to eradicate biofilms once they have formed. The process of biofilm formation takes place in several stages, and the time it takes varies between individual species and even between strains. In the case of *A. lannensis*, previous studies had shown that mature biofilms formed after 6–7 days under laboratory conditions [5]. As observed by many other authors [39,40,41,42], biofilm may be difficult to eradicate. Often, in MICs that are determined for planktonic cells, compounds are very weak or inactive against bacterial cells in biofilms.

**Figure 4:**
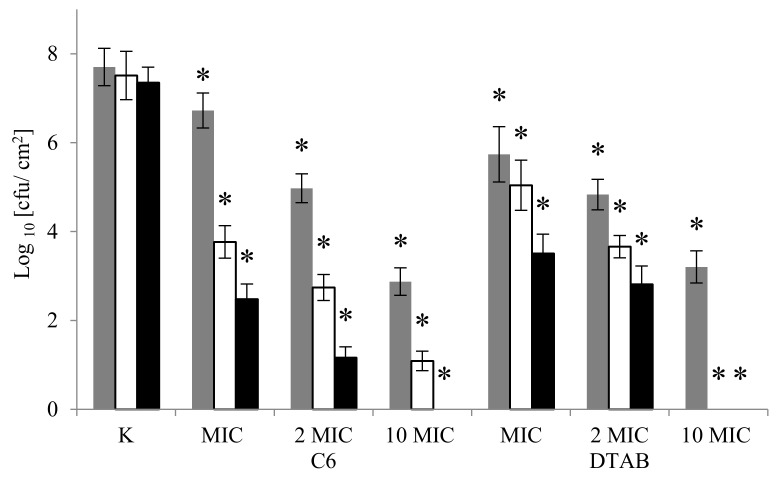
Effect of gemini C6 (**a**) and monomeric DTAB (**b**) surfactants on eradication of biofilm formed by *A. lannensis* FMW1. The results are presented after 1 h (dark grey bar), 4 h (white bar) and 24 h (black bar) treatment by surfactants in the concentration MIC, 2 MIC and 10 MIC, and compared to the control sample (K) without surfactants. * Reduced value of log _10_ differed significantly from the control without surfactant (K).

We investigated the ability of DTAB and C6 to eradicate mature biofilms that are formed on polypropylene. Three concentrations were tested: MIC (determined for planktonic cells), 2 MIC, and 10 MIC (Figure 4). For both of the surfactants, regardless of the concentration used, a reduction in the number of viable cells in the biofilms was observed (*p* < 0.05). After 1 h, gemini surfactant at MIC (0.0036 μM/mL), 2 MIC and 10 MIC decreased cell numbers by 1 log, 2.7 log and 4.8 log, respectively. After 24 h of incubation, samples treated with gemini C6 at 10 MIC showed a total inhibition of bacterial growth. Cabo et al. used BAC to eradicate *S. aureus* biofilm formed on polypropylene [43]. Total eradication was observed after 10 min with a BAC concentration of 0.01 mg/mL. In our study, a reduction of 91.8% in the number of bacterial cells was observed after 4 h of treatment with gemini surfactant at a concentration of 0.024 mg/mL (10 MIC). After 24 h, 100% eradication was achieved. A similar effect was observed after 4 h of treatment with monomeric surfactant, but at the higher concentration of 20.268 μM/mL (6.25 mg/mL). It should be taken into account that the biofilm eradicated in the study by Cabo et al. was created by Gram-positive bacteria *S. aureus* bacteria over 14 h, while in our work mature, six-day old structures that were formed by Gram-negative cells of *A. lannensis* were used [43].

After 24 h of incubation, the greatest reduction in cell numbers was observed with surfactant C6 at MIC and 2 MIC, which reduced cell numbers by 4.2 log and 3.8 log, respectively. These values were almost double those that were observed with monomeric compound (2.2 log for MIC, 2.0 log for 2 MIC). As has been mentioned, dimeric surfactant at 10 MIC after 1 h reduced the number of viable cells in biofilm by 4.8 log. Campanac et al. tested the influence of benzalkonium chloride (BAC_12_) with a C12 alkyl chain on the eradication of biofilms created by *Pseudomonas aeruginosa* and *Staphylococcus aureus* [44]. After 5 min at a concentration of 1 μM/mL, the inhibition of cell growth in biofilm formed over 65 h by *P. aeruginosa* was around 6 log, but at a concentration of 0.1 μM/mL it was only 1 log. Benzalkonium chloride at a concentration of 1 μM/mL weakly inhibited biofilm formed by *S. aureus* cells, by 1 log.

The crucial factors affecting the effectiveness of disinfectants are the concentration of the disinfectant (c) and the contact time (t). The product of these two values for a particular biocide is a constant, i.e., c^ŋ^*t= const [45,46,47]. The ŋ value is a parameter that is determined empirically for each active substance. For quaternary ammonium salts, the value of ŋ equals 1. In other words, if we reduce the concentration value by half, the duration of the required contact time will double. That is to say, if the duration of action by gemini (C6) is reduced to 5 min, a concentration of 0.043 μM/mL should be used. This value is more than twice as low as that reported by Campanac et al. for benzalkonium chloride BAC_12_ [44].

Several parameters influence the complex process of biofilm formation. These include the morphology and physiological state of the bacterial cells, including their hydrophobicity. Even within a single species, the ability of cells to form biofilms can vary [31,36]. A second issue is the aging of biofilms. Most researchers have investigated the activity of antimicrobial agents against young bacterial biofilms, created over 24 h [38,43,48] or 48–72 h [49,50]. Only some studies have used biofilms created over several days [32,36,39,51]. It is well known that older biofilms are more resistant than younger ones to biocides [52]. The third significant parameter is a type of surface. Microorganisms adhere in different ways and in varying quantities to 96-well polystyrene microplates, steel, polymers, or glass. Even with regard to polymers, it is important to distinguish between polypropylene [51], HDPE [38], polyethylene [53], and polystyrene [33]. Surface roughness makes it easier or more difficult for microbial populations to settle. A final important parameter is the environment in which the biofilm forms, including temperature, oxygen availability, pH, and chemical character [39,40,54]. Any antimicrobial agents that are used to inhibit the growth of planctonic cells may also have a disastrous effect on biofilm formation. For this reason, when using synthetic biocides, it is very important to determine their chemical structure accurately.

**Figure 5:**
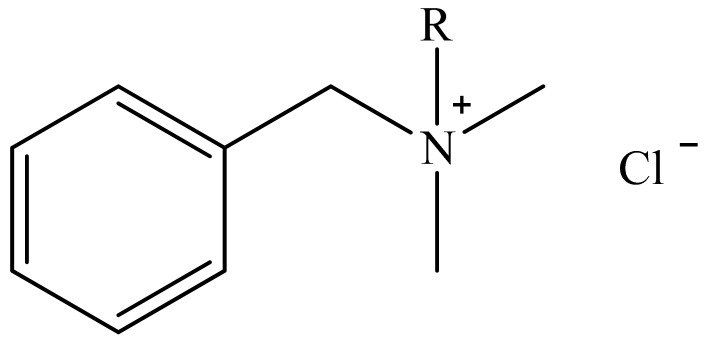
Chemical structure of benzalkonium chloride (*N*-alkyldimethylbenzylammonium chloride) BAC.

In many works, benzalkonium chloride/BAC is used as an antimicrobial or antibiofilm agent [31,39,43,51,55,56,57]. This substance is commercially available and is similar to the active substances that are often used in industrial disinfectants. However, BAC is not a single compound but the generic name for compounds with the chemical structure is presented in Figure 5.

“R” is an alkyl chain, the length of which may vary from C_8_H_17_ to C_18_H_37_. Yet, references to ‘benzalkonium chloride’ in the literature provide no information about the chain length, as have also noted [13]. This is important, as demonstrated in studies by Campanac et al. and El Hage et al. who showed how the MIC value and the viability of different species of bacteria (*P. aeruginosa*, *S. aureus*, *E. coli*) and microscopic fungi (*Candida albicans*, *Aspergillus niger*) changed when BACs with different alkyl chain lengths were used [44,58]. For instance, the greatest reduction in cfu for *P. aeruginosa* and *E. coli* occurred when the 12-carbon alkyl compound was present [44]. Commercially available benzalkonium chlorides used for disinfection are often a mixture of 2–3 ingredients. One of the compounds that was used in a study by Massi et al. against *P. aeruginosa* was a commercially available mixture of BACs containing 50% C_14_H_29_, 40% C_12_H_25_, and 10% C_10_H_21_ [59].

Just as important as the cationic group in the BAC molecule, is the anion. For benzalkonium chloride this is usually chlorine, but Br^−^ or I^−^ can be also used. It means that MIC values for commercial benzalkonium halides can vary in a wide range [58]. When examining the impact of compounds such as mono or gemini surfactants containing long alkyl chain, it is important to take into account a “cut-off” effect. This phenomenon is described in current literature [60,61,62]. It shows, that for a particular length of alkyl chain, the biological activity cannot increase further. In other words, MIC values increase, and therefore, compounds with longer alkyl chains are not as effective as their shorter analogs.

In conclusion, it is recommended for scientific purposes to use compounds with strictly defined chemical structures, whether for studies on planktonic cells, biofilm formation, or biofilm eradication.

## 3. Materials and Methods

### 3.1. Microorganisms and Technical Materials

The experiments were performed with *A. lannensis* FMW1, isolated from strawberry-flavoured mineral water in Poland. The biological material was stored in liquid GC medium (0.3% peptone (*w*/*v*), 0.3% yeast extract (*w*/*v*), 0.7% CaCO_3_ (*w*/*v*)) with 2% glucose (*w*/*v*) at 4 °C.

Rectangular pieces of polypropylene (PP) 70 × 25 mm (Packor Packaging, Skierniewice, Poland) were used for adhesion tests. The test material was certified by the Polish National Institute of Public Health and approved for contact with food [5].

### 3.2. Antimicrobial Agents

The antimicrobial agents used were n-dodecyltrimethylammonium bromide (DTAB), monomeric alkylammonioum salt and hexamethylene-1,6-bis-(*N,N*-dimethyl-*N*-dodecylammonium bromide) (C6) dimeric alkylammonium salt. Dodecyltrimethylammonium bromide is commercially available (Aldrich, Munich, Germany), while the gemini surfactant (C6) was synthesized by the reaction of *N*,*N*-dimethyl-*N*-dodecylamine (36.4 g, 0.18 M) (Aldrich, Munich, Germany) with 1,6-dibromohexane (21.4 g, 0.08 M) (Aldrich, Munich, Germany) in acetonitrile (120 mL) under reflux for 6 h, according to a procedure described in literature [19]. The crude product was crystallized from acetonitrile to give white crystals of C6 (Yield 90.4%, m.p. 231–232 °C; elemental analysis: found (calc.) %C 60.51 (60.88); %H 11.65 (11.12); %N 4.09 (4.18); ES + MS *m*/*z* 255 (C34H74N2/2).

### 3.3. Minimal Inhibitory Concentration

The MIC values for the bacteria were determined using the tube standard twofold dilution method, as described by Brycki, Kowalczyk & Koziróg [19]. Incubation was conducted on a rotary shaker (100 rpm) for 24 h at 28 °C in Triptic Soy Broth medium (TSB; Merck,Darmstad, Germany) with 2% glucose. The bacterial culture was centrifuged (8000 rpm for 10 min) and suspended in salt solution to obtain a cell level of 10^6^ cfu/mL. One mL of cell suspension was mixed with 1 mL of medium with serial dilutions of the tested compounds. The medium consisted of TSB with 2% glucose (*w*/*v*), minimal medium (0.1% K_2_HPO_4_ (*w*/*v*), 0.1% KH_2_PO_4_ (*w*/*v*), 0.3% yeast extract (*w*/*v*)), and 2% of one of the carbon sources: glucose, sucrose, fructose, or maltose. The samples were incubated at 28 °C for 24 h. Bacterial suspensions in culture media without the tested biocides were used as control samples. The MICs were defined as the lowest concentrations of the compounds in which there was no visible growth of *Asaia* sp.

### 3.4. Viability of A. lannensis Cells in the Presence of Microbiocides

Bacterial growth was evaluated in liquid medium 16 mL TSB + 2% glucose with the addition of 2 mL monomeric DTAB or gemini surfactant C6, at ¼ MIC, ½ MIC or MIC. Each test flask was inoculated with 2 mL of standardized bacterial suspension to obtain a bacterial cell level of approximately 10^7^ cfu/mL. The samples were incubated at 25 °C on a laboratory shaker (100 rpm) for six days. After 1, 4, 8, 12, 24, 48, 72, 96, and 144 h, 1 mL of each mixture was transferred to 9 mL of 0.85% (*w*/*v*) saline with a mixture of neutralizers (5% Tween 80, 2% lecithin and 0.5% sodium thiosulfate) [44]. Viable cells were determined on TSA agar medium (Merck, Darmstad, Germany), supplemented with 2% glucose using the conventional plate count method. After incubation at 25 °C for 48 h, the colonies of *A. lannensis* were counted.

### 3.5. Adhesion Analysis

#### 3.5.1. Short-Term Contact with Antimicrobial Agents

After 1 h of contact between the bacterial cells with surfactant, under the conditions described above, the biomass was centrifuged (8000 rpm, 10 min). The supernatant was removed and the biomass was washed for 10 min with saline and neutralizers, and then suspended in saline. The control was a sample without antimicrobial agents. To each test flask were added 18 mL TSB + 2% glucose, sterile rectangular polypropylene disks (60 by 25 mm) placed vertically and 2 mL of bacterial suspension (10^6^–10^7^ cfu/mL). The samples were incubated at 25 °C on a laboratory shaker (100 rpm) for six days. After three and six days, the PP plates were removed from the culture media, rinsed with sterile distilled water and swabbed using sterile swabs. The bacterial suspensions were vortexed vigorously in 0.85% (*w*/*v*) saline with 0.1% (*v*/*v*) Tween 80, and the dilutions were transferred onto TSA + 2% glucose medium. After incubation at 25 °C for 48 h, the colonies of bacteria were counted and the numbers of attached cells were determined (cfu per square centimetre).

#### 3.5.2. Adhesion in the Presence of Antimicrobial Agents

The adhesion ability of the bacterial strains was evaluated according to the methods described by Kręgiel [5]. To each flask were added: 16 mL TSB + 2% glucose, polypropylene disks, 2 mL of bacterial suspension (10^6^–10^7^ cfu/mL), and 2 mL of one of the surfactants at ¼ MIC, ½ MIC, or MIC. In the control sample, instead of the antimicrobial agents, 2 mL of sterile distilled water was added. The samples were incubated at 25 °C on a laboratory shaker (100 rpm) for six days. After three and six days, the plates were removed from the culture media and were placed in 20 mL of saline with neutralizers for 10 min. The PP surfaces were then rinsed with sterile distilled water and swabbed using sterile swabs for surface testing. To determine the number of viable bacterial cells, both on the tested surfaces and in the culture media, the plate count method was used, as described above. The results were expressed as cfu per square centimetre.

### 3.6. Biofilm Eradication

Biofilm was prepared in in flasks containing 16 mL TSB + 2% glucose, polypropylene disks, and 2 mL of bacterial suspension (10^6^–10^7^ cfu/mL). The control was a sample without antimicrobial agents. The samples were incubated at 25 °C on a laboratory shaker (100 rpm) for six days. The PP plates were then removed from the culture medium, rinsed with sterile distilled water, and transferred to flasks containing 18 mL PBS buffer and 2 mL monomeric or dimeric surfactant at MIC, 2 MIC, or 10 MIC. A control sample was transferred to PBS buffer without antimicrobial agents. After 1, 4, and 24 h, the PP plates were removed from the culture media and were placed in 20 mL of saline with neutralizers for 10 min. The PP material was then rinsed with sterile distilled water and swabbed using sterile swabs for surface testing. The number of cells in the biofilm was determined using the plate count method. The results were expressed in cfu per square centimetre.

### 3.7. Statistics

The mean results from three independent experiments were calculated, together with their standard deviations. Statistical differences in the data were compared using a one-way repeated measures analysis of variance (ANOVA; OriginPro 9.2.214, OriginLab Corp., Northampton, MA, USA). Statistical significance was set at 5% (*p* < 0.05).

## 4. Conclusions

In this study, hexamethylene-1,6-bis-(*N*,*N*-dimethyl-*N*-dodecylammonium bromide) (C6) belonging to the group of gemini surfactants has been shown to exhibit high antimicrobial efficacy against the acetic acid bacteria *A. lannensis—*a frequent contaminant in the beverage industry. Even at a very low concentrations of 10^−2^–10^−3^ μM, this compound not only reduced the number of viable bacterial cells in the planktonic state and prevented cell adhesion to a polypropylene test surface, but also appeared to be effective at eradicating biofilm. These results suggest that hexamethylene- 1,6-bis-(*N,N*-dimethyl-*N*-dodecylammonium bromide) (C6) could have a wide range of practical applications as an active compound. In particular, gemini surfactant C6 has potential for application in the soft drinks industry, where *Asaia* spp. constitute the one of major bacterial contaminants of final products.

## Figures and Tables

**Table 1:** The value of of minimal inhibitory concentration of monomeric *n*-dodecyltrimethylammonium bromide (DTAB) and gemini (C6) surfactants against *A. lannensis* FMW1 strain.

Type of Culture Medium	MIC [μM]	
	DTAB	C6
Minimal medium + 2% glucose	0.1267	0.0073
Minimal medium + 2% saccharose	0.2534	0.0073
Minimal medium + 2% fructose	1.0134	0.0073
Minimal medium + 2% maltose	2.0268	0.0073
TSB + 2% glucose	2.0268	0.0036

## Supplementary Materials

Supplementary materials are available online. Table S1: The inhibition of *Asaia lannensis* cells forming biofilms [%] in the presence of monomeric (DTAB) or gemini (C6) surfactants.
