# Supplementary material for: Action of Monomeric/Gemini Surfactants on Free Cells and Biofilm of Asaia lannensis
Source: Molecules. 2017 Nov 22;22(11):2036. doi: 10.3390/molecules22112036 (PMC6150408; doi:10.3390/molecules22112036)
Supplement: Supplementary file 1 [file molecules-22-02036-s001.pdf]

Supplementary Table S1

Table S1. The inhibition of *Asaia lannensis* cells forming biofilms [%] in the presence of monomeric (DTAB) or gemini (C6) surfactants.

Time of action [days]	Type of surfactant	Surfactant concentration [μM]		
		**¼ MIC**	**½ MIC**	**MIC**
3	C6	63.2	100	100
	DTAB	23.2	44.6	80.9
6	C6	48.8	100	100
	DTAB	24.3	59.8	93

% inhibition = 100 - [(log_{10} with surfactant/ log_{10} without surfactant)*100] [33]
